# Estrogen Receptor Type 1 and Type 2 Presence in Paravertebral Skeletal Muscles: Expression Level and Relation to Phenotype in Children with Idiopathic Scoliosis

**DOI:** 10.3390/genes13050739

**Published:** 2022-04-22

**Authors:** Tomasz Kotwicki, Marek Tomaszewski, Mirosław Andrusiewicz, Aleksandra Śliwa, Błażej Rusin, Małgorzata Kotwicka

**Affiliations:** 1 Department of Spine Disorders and Pediatric Orthopedics, Faculty of Medicine, Poznan University of Medical Sciences, 28 Czerwca 1956 r. Street 135/147, 61-545 Poznan, Poland; kotwicki@ump.edu.pl (T.K.); mtooma@wp.pl (M.T.); blazej.rusin@gmail.com (B.R.); 2 Chair and Department of Cell Biology, Faculty of Health Sciences, Poznan University of Medical Sciences, Rokietnicka 5D, 60-806 Poznan, Poland; andrus@ump.edu.pl (M.A.); glodek@ump.edu.pl (A.Ś.); 3 Halluxmed Surgery of the Foot/Knee, Komornicka 57, 62-052 Komorniki, Poland

**Keywords:** estrogen receptor 1, estrogen receptor 2, paravertebral skeletal muscles, idiopathic scoliosis, scoliosis angle, scoliosis progression

## Abstract

The study aimed to detect the presence and assess the expression levels of the estrogen receptors type 1 (ESR1) and type 2 (ESR2) within paravertebral skeletal muscles of female patients with idiopathic scoliosis (IS) in relation to phenotype parameters. Intraoperatively, the muscle samples were obtained from 35 adolescent females. The RT-qPCR, western blot and immunohistochemistry techniques were applied. The ESR1 and ESR2 were detected within paravertebral skeletal muscle cells, either the superficial or the deep ones. The *ESR1* expression level was significantly higher in the deep muscles compared to the superficial ones. A left-right asymmetry of the *ESR1* and *ESR2* expression level was demonstrated in the deep muscles. There was a significant relationship between the expression asymmetry and either the Cobb angle or the progression risk factor: both parameters decreased to the smallest values in the case of symmetric *ESR1* or *ESR2* expression, while they increased with increasing expression asymmetry. In conclusion, the ESR1 and ESR2 presence was confirmed in skeletal paravertebral muscles of patients with idiopathic scoliosis. The increased expression level and asymmetry of estrogen receptors in deep skeletal muscles was related to increasing scoliotic deformity magnitude or increasing risk of deformity deterioration. These findings may highlight the etiopathogenesis of IS in children.

## 1. Introduction

Idiopathic scoliosis (IS) is the most frequent developmental deformity of the spine in children and affects 2–3% of the general population [1]. The IS definition formulated by the Scoliosis Research Society (SRS) comprises a lateral curvature of the spine with vertebral axial rotation, presenting the Cobb angle of at least 10°, as measured on the anteroposterior radiograph made in a standing position [1].

A three-dimensional scoliotic deformity manifests with a sagittal plane disorder of the physiological thoracic kyphosis or lumbar lordosis, a lateral inflection in the frontal plane, and axial vertebrae rotation in the horizontal plane. The IS prevalence in females is two times higher in boys when the Cobb angle is 10° to 30°, however, with the angle of more than 30°, the ratio increases to 8:1 in favor of girls who are more prone to scoliosis deterioration during adolescence [1].

So far, the IS pathogenesis has not been explained. There are numerous hypotheses pointing to the origins of the deformity in the genetic [2,3,4], biochemical, and biomechanical, including relative anterior spine overgrowth, neurohormonal, bone, and muscular factors [5]. To keep an upright posture, constant coordination between the central nervous system and the skeletal and muscular systems is required, so dysfunction and/or improper communication between them may result in the IS occurrence [5]. Stokes proposed a vicious circle hypothesis for scoliosis curve progression: continuous asymmetrical loading of the spine beyond a given threshold value triggers curve progression [6].

Researchers have postulated that in the paravertebral muscles, the asymmetric expression of the melatonin receptor 1A/B, calmodulin, and nerve growth factor [7] may be involved in IS pathogenesis. It remains unclear whether the observed asymmetry in the expression of the aforementioned factors represents the primary causative factor or a secondary phenomenon induced by the asymmetry of the forces exerted over the vertebral column.

Estrogen receptor 1 (ESR1) and estrogen receptor 2 (ESR2) were shown to be expressed unevenly in various tissues. Both receptor forms can be present together within the same tissue. However, there exist tissues wherein one receptor presents a higher expression level than the other. Few studies have described the estrogen receptors (ESRs) expression in human skeletal muscle. Lemoine et al. identified *ESR1* at the mRNA level in the pectoral and the deltoid muscles [8]. Wiik et al. localized ESR1 in the vastus lateralis muscle, both on mRNA and protein levels [9]. Later on, a similar observation was made for the ESR1 and ESR2 in skeletal muscles, both in male and female patients [10]. Few other studies have investigated the expression of ESR1 and ESR2 in deep paravertebral muscles of IS patients. Kudo et al. described asymmetry of *ESR1* expression in the paravertebral muscles [7]. On the other hand, Zamecnik et al. did not confirm the asymmetry of *ESR2* expression [11,12].

It is postulated that estrogen affects the strength of skeletal muscle. It was shown in meta-analysis studies that estrogen hormone replacement therapy had a significant effect on muscle force in postmenopausal women. The mechanism for estrogen action in most tissues is mediated through estrogen receptors that appear to modify muscle force at the molecular level [13,14]. Modulation of the estrogen receptors’ action could be determined by their expression level and both epigenetic and genetic alterations. Our previous studies showed that, e.g., differences in methylation levels in estrogen receptor genes may be associated with IS etiopathogenesis and may affect the tissue-dependent response to estrogens [15,16]. Moreover, many promising polymorphisms within different genes (including *ESR1*, *ESR2*) have been identified for the occurrence and curve progression of idiopathic scoliosis [17]. The SNPs identified in ESR1 could be associated with, e.g., sarcopenia characterized by loss of muscle mass and strength [18] or protection against muscle injury by lowering muscle stiffness [19]. Little is known about *ESR2* genetic alterations concerning skeletal muscle tissue function, which is impaired in idiopathic scoliosis. The *ESR1* expression might be related to the muscular load on either side of scoliosis.

Our previous studies focused on the *ESR2* gene polymorphism in patients with idiopathic scoliosis [20], as well as on methylation of both *ESR1* and *ESR2* genes [15,16]. The methylation pattern of CpG sites in the regulatory regions of the *ESR2* gene in the deep paraspinal muscle tissue was associated with either the occurrence but not with the severity of IS. The difference in *ESR1* T-DMR2 (tissue dependent and differentially methylated region 2) CpGs methylation of the deep paravertebral muscles on the concave side of the curvature may be associated with IS severity. We also investigated the presence of estrogen receptor coactivator proline glutamic acid and leucine-rich protein in deep paravertebral muscles of patients with idiopathic scoliosis [21].

Estrogens influence skeletal muscle activity on several levels. Estrogens regulate vascular-endothelial growth factor production. The factor stimulates angiogenesis, cell differentiation and anti-apoptotic action in muscle cells [22]. They participate in nitric oxide production, modulating muscle repair, and adaptation to physical training [23]. Estrogens regulate oxidative metabolism within skeletal muscle by shifting substrate metabolism and reducing liver and muscle glycogen usage [24].

Analyzes concerning the concentration level of circulating estrogens are inconsistent. A study performed on adult females with IS revealed decreased circulating estradiol levels compared to controls [25]. However, Raczkowski found no difference in estradiol concentrations [26]. In 2015, Kulis et al. stated that in both pre- and post-menarchial scoliotic girls, estradiol levels were lower than pre- and post-menarchial controls [27]. The uncertain data led to the hypothesis that ESR abnormalities (in structure, location, or quantity) could be the reason for inappropriate muscular tissue response to estrogens.

The study aimed to investigate whether ESR1 or/and ESR2 are present in the paravertebral muscles of IS patients at both mRNA and protein levels. Moreover, the relation of the ESRs’ gene expression versus scoliosis radiological parameters was investigated.

## 2. Materials and Methods

### 2.1. Patients

Thirty-five Caucasian girls from one Central European country (Poland) underwent surgical scoliosis correction at the Department of Spine Disorders and Pediatric Orthopedics of Poznan University of Medical Science. The inclusion criteria were: diagnosis of IS, indication for surgical scoliosis correction, and informed consent. Out of 35 patients, there were 12 Adolescent Idiopathic Scoliosis (AIS) patients and 23 Early Onset Idiopathic Scoliosis (EOIS) patients. According to the Lenke scoliosis classification, the patients’ mean age was 15 years and 5 months ± 26 months [28]. The patients demonstrated Lenke types as follows, type I: 18 patients, type II: 4, type III: 9, type IV: 1, type VI: 3 patients. The radiological examination was performed using standing AP and lateral long cassette radiographs. The mean Cobb angle value was 74.8° ± 19.1°. The patients underwent radiological and clinical examinations (Table 1).

### 2.2. Radiological Examination

The available radiographs of the spine, which had been routinely performed during scoliosis therapy, were used. No additional radiograph was prescribed. The entire spine-long cassette standing antero-posterior radiographs were used. One parameter was measured: scoliosis angle in degrees by the Cobb method involving the measurement of the angle between the line plotted along the upper vertebral body of the upper-end vertebra and the lower vertebral body of the lower-end vertebra of the spinal curvature.

One additional parameter was calculated: the scoliosis progression risk factor (PRF) according to the formula proposed by Lonstein and Carlson [29]:

(1) $$\mathrm{Progression}\;\mathrm{risk}\;\mathrm{factor}\;\left(\mathrm{PRF}\right)=\frac{\mathrm{Cobb}\;\mathrm{angle}\;-3\times \mathrm{Risser}\;\mathrm{sign}}{\mathrm{calendar}\;\mathrm{age}}$$

The Risser sign was assessed in a regular way from 0 to 5.

### 2.3. Muscle Tissue Samples

Muscle tissue samples were obtained intraoperatively from the following skeletal muscles: (1) concave deep paravertebral, (2) convex deep paravertebral, (3) superficial; the samples were obtained at the apical level of the primary scoliotic curvature.

Each skeletal muscle sample was split into three fragments: (1) material for RT-qPCR was immersed in RNase inhibitor (RNALater; Sigma; Saint Louis, MO, USA) and stored at −80 °C until mRNA isolation, (2) material for immunohistochemistry was immediately fixed in 4% paraformaldehyde (Chempur; Piekary Śląskie, Poland), and (3) material for protein isolation was placed in phosphate buffered saline (PBS, Chempur; Piekary Ślaskie, Poland) and stored at −80 °C until further procedures.

### 2.4. Isolation of Total Cellular RNA

Muscle tissue samples (~100 mg) were snap-frozen and homogenized using a mortar and a pestle. The obtained tissue powder was used for total RNA extraction using TriPure Isolation Reagent (Roche; Basel, Switzerland) according to the manufacturer with a modification of RNA precipitation step. The RNA was precipitated with 600 µL of isopropanol at −80 °C for 10 min instead of 500 µL of isopropanol at room temperature. The TriPure isolation protocol was carried out twice for each tissue sample.

The quantity and purity of total RNA were analyzed spectrophotometrically using the NanoDrop ND-1000 spectrophotometer (Thermo Fisher Scientific, Waltham, MA, USA). The integrity of the RNA was evaluated by denaturing formaldehyde agarose gel electrophoresis in FA buffer (20 mM MOPS, 5 mM sodium acetate, 1 mM EDTA, 200 mM paraformaldehyde; pH 7.0; Sigma-Aldrich; Saint Louis, MO, USA). One microliter of isolated RNA was separated in the presence of ethidium bromide, and gels were visualized with G:BOX imaging system.

### 2.5. Reverse Transcription and Quantitative mRNA Assessment

The reverse transcription reaction (RT) was conducted using Transcriptor Reverse Transcriptase according to the manufacturer’s protocol (Roche; Basel, Switzerland) in a total volume of 20 µL. One microgram of isolated total RNA with 5 mM oligo(d)T10 primer (Genomed; Warsaw, Poland) in RNase/DNase/pyrogen-free water (Thermo Fisher Scientific, Waltham, MA, USA) was denatured 10 min at 65 °C. Samples were chilled on ice. The reaction mixture was then supplemented with 5 U ribonuclease inhibitor (RNasin, Roche; Basel, Switzerland), 10 U of Transcriptor Reverse Transcriptase (Roche), 10 mM dNTPs (Novayzm; Poznan, Poland) and 1× reaction buffer (Roche; Basel, Switzerland). The thermal profile of cDNA synthesis was as follow: 10 min at 25 °C, 30 minutes at 55 °C, and 5 min at 85 °C. cDNA was stored in −20 °C until further analysis but no longer than seven days.

Subsequently, cDNA was used in a quantitative polymerase chain reaction (qPCR) with LightCycler 2.0 carousel glass capillary-based system (Roche, Manheim, Germany). All reactions were made in duplicates using independently synthesized cDNA.

### 2.6. Quantitative Polymerase Chain Reaction

Quantitative Polymerase Chain Reaction (qPCR) assays were designed to cover all splice variants of analyzed genes. In the case of *ESR1* (GenBank: NM_000125.3; NM_001122740.1; NM_001122741.1; NM_001122742.1) the qPCR was conducted with TaqMan Hydrolysis Probes (Roche; Basel, Switzerland). The probes were selected with ProbeFinder software v. 2.50 (available at https://lifescience.roche.com/shop/en/us/overviews/brand/universal-probe-library; last accessed on 28 September 2017; discontinued). Human ready to use *HPRT* Reference Gene Assay (assay No 05046157001; Roche; Basel, Switzerland) was used as an internal control and relative expression level assessment.

The final concentration of reagents in assays in the total volume of 20 µL reaction mix was used to quantify as follows: 1× FastStart TaqMan Probe Master Mix (Roche; Manheim, Germany), 5 µL cDNA, 0.5 mM of each primer set (Genomed; Warsaw, Poland) and 0.1 mM GOI hydrolysis probe or 0.4 mM reference gene Probe (Roche; Manheim, Germany).

The *ESR2* expression (GeneBank: NM_001437; NM_001040275; NM_001040276) was quantified using SybrGreen I. In this assay, expression of the *HPRT* reference gene (GenBank NM_000194.2) was measured using the same method. Primers for qPCRs with SybrGreen I were designed using Primer3Plus software v.2.3.6 (http://primer3plus.com, last accessed on 12 January 2012) and verified using BLAST alignment.

In SybrGreen I assays, the reaction mix contained: 1× Light Cycler FastStart DNA Master SybrGreen I (Roche; Basel, Switzerland), 3 mM Mg^2+^ solution, 3 µL cDNA, 0.5 mM of each primer (Genomed, Warsaw, Poland) in the total volume of 20 µL. After amplification, the identity of the *ESR2* and *HPRT* products was confirmed by melting curves analyses.

Primers and probes used in qPCR are presented in Table 2. The thermal profiles of the reactions are shown in Table 3.

Reaction efficiencies were derived from qPCR reactions performed using decimal dilutions of RT-qPCR products purified with DNA Clean & Concentrator kit (Zymo Research; Irvine, CA, USA). The expression level was normalized against standard curves and *HPRT* reference gene using LightCycler 4.05 software. The relative expression level of *ERS*s (concentration ratio) was used for statistical analyses.

Randomly selected *ESR2* qPCR products were sequenced to confirm the specificity of amplification. As the *HPRT* reference gene was amplified in a previously validated SybrGreen assay and with a commercially available *HPRT* assay, verification of the qPCR product was not required.

### 2.7. Protein Concentration and Western Blot Analyses

Tissue samples immersed in PBS were used for protein isolation and western blot analyses to confirm the protein presence and identity. After mechanical homogenization, tissue samples were suspended in an isolation buffer containing: 320 mmol/L sucrose, 10 mM/L Tris, 1 mM/L Mg^2+^, 2 mM/L dithiothreitol, 0.2 mM/L protease inhibitor PMSF; pH 7.5 and centrifuged (10 min, 3000× *g*, 4 °C). The supernatant was transferred into fresh tubes and again centrifuged (10 min, 3 000× *g*, 4 °C); supernatant containing purified proteins was used in further analyses. According to the manufacturer’s protocol, protein concentration was assessed according to the Bradford assay using Quick Start Bradford Dye Reagent (Bio-Rad; Hercules, CA, USA). The results were compared to standard decimal dilutions of bovine serum albumin fraction V (Roche; Basel, Switzerland), ranging from 1 mg/mL to 1 µg/mL. The protein analyses of the estrogen receptors were performed using denaturing polyacrylamide gel electrophoresis (SDS-PAGE) followed by immunoblotting as described before [30]. Rabbit polyclonal antibodies against ESR1 (sc-8005, Santa Cruz Biotechnology, Dallas, TX, USA), ESR2 (ab3576, Abcam; Cambridge, UK), and GAPDH (sc-25778, Santa Cruz Biotechnology) were used in the following dilutions: 1:500, 1:250, and 1:1000, respectively. The secondary polyclonal goat anti-rabbit antibody linked with horseradish peroxidase was used in a dilution of 1:2500 in blocking buffer. Immunoreactive bands were visualized on membranes using EZ-ECL (Biological Industries; Beit-Haemek, Israel) according to the manufacturer’s protocol. Bands’ optical density was analyzed with G:BOX imaging system and GeneSys software (Syngene; Cambridge, UK).

### 2.8. Immunohistochemical Localization of ESR1 and ESR2 Proteins

The analysis was performed on 10 randomly selected patients. Tissue, fixed in 4% paraformaldehyde and embedded in paraffin, was cut into 3 µm tissue sections, placed on the silanized glass slides, dewaxed, and rehydrated. Antigens were retrieved by microwave heating in sodium citrate buffer (10 mM Sodium Citrate, 0.05% Tween 20, pH 6.0, Sigma-Aldrich; Saint Louis, MO, USA).

To block endogenous peroxidase activity, tissue sections were treated with 3% hydrogen peroxide for 10 min at room temperature and subsequently incubated in blocking buffer (100 mM Tris-HCl, 0.9% NaCl, 0.05% Tween-20, 3% BSA; pH 7.5). In the next step, slides were incubated in a humid chamber overnight at 4 °C with primary polyclonal anti-ESR1 and anti-ESR2 (the same as used in the case of western blot analysis) diluted in a ratio of 1:50 in blocking buffer. After incubation, slides were rinsed three times in TBS buffer (50 mM Tris-Cl, pH 7.6; 150 mM NaCl).

The location of ESR1 and ESR2 antigens in analyzed tissue sections was visualized with EnVision™+Dual Link System-HRP (Dako, Glostrup, Denmark) according to the manufacturer’s protocol and analyzed using a light microscope. Results of microscopic observations were digitalized.

Archival endometrial tissue samples with known ESRs expression were used as a positive control in these experiments. Negative control experiments included reactions in which a nonimmune serum replaced primary antibodies.

### 2.9. Asymmetry of ESRs Gene Expression

In 35 girls with IS, the level of ESRs gene expression was assessed for the deep concave (*ESR1_cnc_*, *ESR2_cnc_*), and deep convex (*ESR1_cvx_*, *ESR2_cvx_*) muscles.

The ratio (convex to concave) of gene expression level was calculated for each receptor using the formulas:

(2) $$r_{ESR1}=\frac{ESR1_{cvx}}{ESR1_{cnc}}{\;r}_{ESR2}=\frac{ESR2_{cvx}}{ESR2_{cnc}}$$

The correlation of the *rESR1* or *rESR2* gene expression level versus the Cobb angle and versus the Progression Risk Factor was studied.

### 2.10. Statistical Analyses

Data analyses were performed using Statistica 10.0 software (StatSoft Inc., Tulsa, OK, USA). Kolmogorov–Smirnov test was applied to check for data normality and the Fisher test was used for variance heterogeneity analysis. The Friedman test and multiple comparison tests were applied. The Pearson correlation coefficient or the Spearman test was used to assess the correlations. *p* = 0.05 was considered significant.

## 3. Results

The Cobb angle of the primary curvature ranged from 50° to 114°, mean 74.8° ± 19.1°. The Progression Risk Factor ranged from 1.6 to 8.5, mean 4.5 ± 2.1. For six patients, the PRF value was below 2.5, which according to Lonstein and Carlson [29], corresponds to an 80–90% risk of scoliosis progression. The remaining patients revealed the PRF value greater than 2.5, corresponding to 100% progression risk.

### 3.1. ESR1 and ESR2 Protein Presence

**Figure 1:**
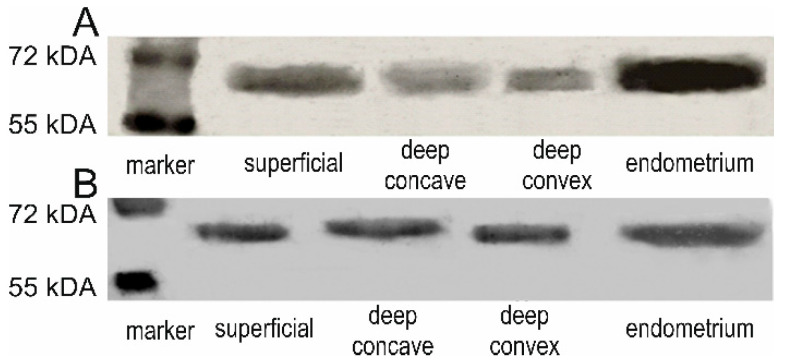
Representative western blot analysis showing the presence of ESR1 (**A**) and ESR2 (**B**) proteins.

**Figure 2:**
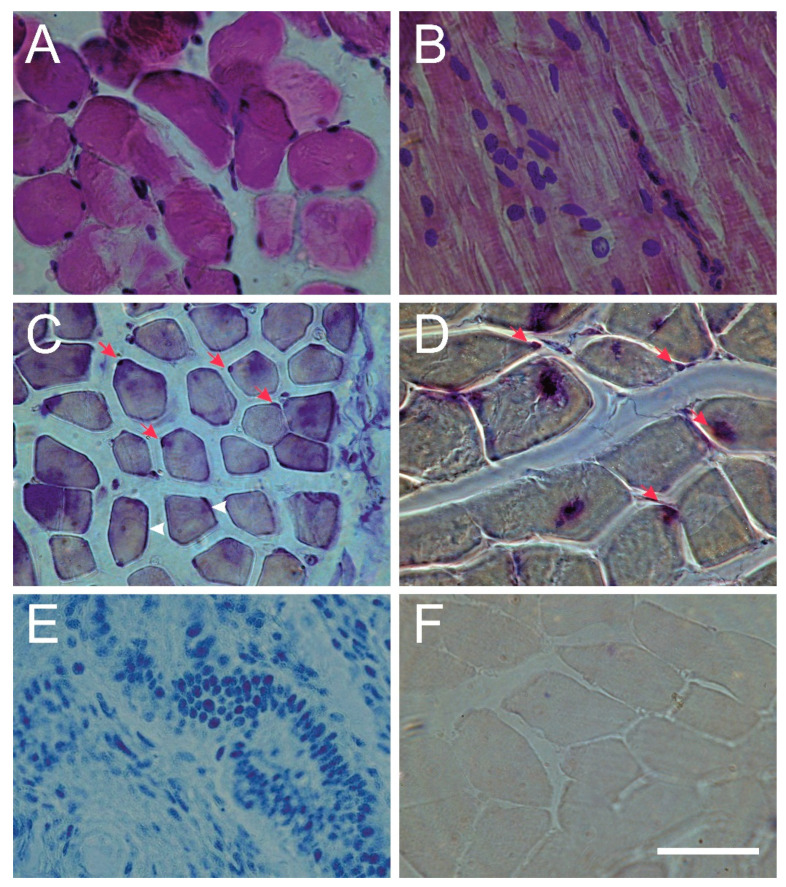
Immunohistochemical examination of the muscle tissue of individual patient. (**A**–**D**) and controls (**E**,**F**). Hematoxylin and eosin staining ((**A**)—cross-section, (**B**)—longitudinal-section; skeletal muscles were identified by peripheral nuclei and large amounts of cytoplasm). Immunolocalization of the ESR1 (**C**) and ESR2 (**D**) presence (purple color). ESR1 and ESR2 were detected in the nuclei of the back muscle cells (red arrows); ESR1 expression was also observed in the muscle cell membrane (white arrowhead). Endometrial control tissue–positive control (**E**), and muscle tissue–negative control (**F**). In the negative control, the primary antibody was omitted. Scale bar = 100 μm.

We confirmed the ESR1 and ESR2 protein presence and identity by western blot analyses in examined samples (Figure 1). Positive immunohistochemical staining for ESR1 and ESR2 was detected in the nuclei of the back muscle cells in all examined samples. The ESR1 expression was also observed in the muscle cell membrane Figure 2.

### 3.2. ESR1 and ESR2 mRNA Expression Levels

Concentration ratio data (referring to relative expression level) of the *ESR1* and *ESR2* is presented in Table 4. The obtained values are standard curves PCR efficiency assessment derivates and the normalization of hypoxanthine-guanine phosphoribosyltransferase reference gene (*HPRT*).

The *ESR1* expression levels differed significantly (*p* = 0.002, Friedman test) among the three muscle groups tested. The *ESR1* expression level was significantly lower in superficial muscles compared to deep concave muscles (*p* < 0.05, multiple comparison test) and compared to deep convex muscles (*p* < 0.01). The concave versus convex *ESR1* expression difference was not significant (*p* > 0.05). The *ESR2* expression level did not differ significantly among the muscle groups (*p* > 0.05, Friedman test).

### 3.3. ESR1 Expression Asymmetry

The convex to concave expression ratio was superior to 1.0 in 18 patients (*rESR1* > 1), which denoted higher *ESR1* expression on the convex side. In comparison, it was inferior to 1.0 in the remaining 17 patients (*rESR1* < 1), which denoted higher *ESR1* expression on the concave side.

### 3.4. ESR2 Expression Asymmetry

The convex to concave expression ratio was superior to 1.0 in 14 patients (*rESR2* > 1), inferior to 1.0 in 14 patients (*rESR2* < 1) and equal to 1.0 in 5 patients (*rESR2* = 1).

### 3.5. ESR1 Expression Level versus Phenotype Parameters

**Figure 3:**
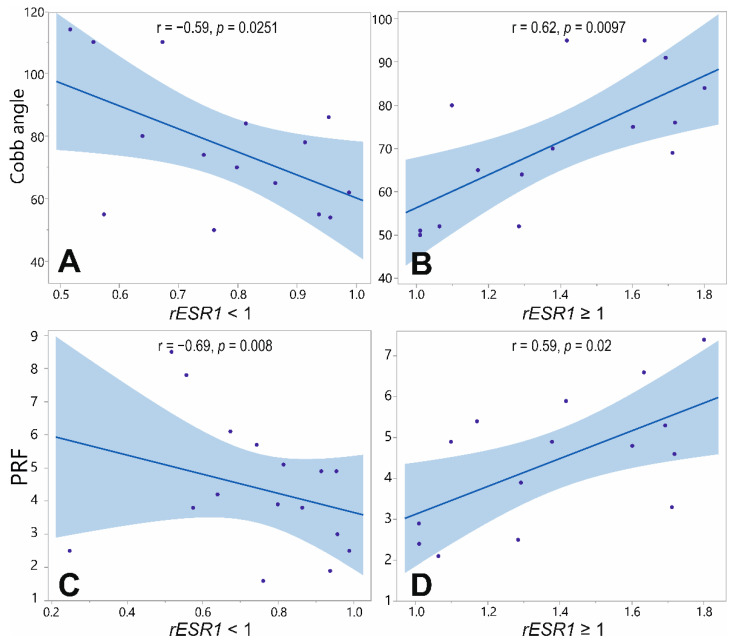
Correlation between ratio (convex to concave) of *ESR1* gene expression level (*rESR1*) in deep paravertebral and phenotype parameters. (**A**). Correlation between the *rESR1* and the value of the Cobb angle in patients with *rESR1* < 1. (**B**). Correlation between the *rESR1* and the value of the Cobb angle in patients with *rESR1* > 1. (**C**). Correlation between the *rESR1* and the value of the risk of progression factor (PRF) in patients with *rESR1* < 1. (**D**). Correlation between the *rESR1* and the value of the PRF in patients with *rESR1* > 1. Linear fit lines and 95% confidence intervals are shown in blue.

No significant correlation between the *ESR1* expression level and the Cobb angle values was found for all patients (*N* = 35), concerning the superficial muscles (*p* = 0.1), the deep concave (*p* = 0.5), nor the deep convex muscles (*p* = 0.7). However, once the patients were divided into subgroups presenting *rESR1* > 1 (*N* = 18) and showing *rESR1* < 1 (*N* = 17), the correlations revealed significance within these subgroups: a positive linear correlation r = 0.62, *p* = 0.0097, and a negative linear correlation r = −0.59, *p* = 0.0251, respectively (Figure 3A,B).

There was no significant correlation between the *ESR1* expression level and the Progression Risk Factor (PRF). However, once the patients were divided as described above (*rESR1* > 1 and *rESR1* < 1), the subgroup analysis revealed a significant correlation: r = 0.59, *p* = 0.02 and r =−0.69, *p* = 0.008, respectively (Figure 3C,D).

### 3.6. ESR2 Expression Level versus Phenotype Parameters

No significant correlation between the *ESR2* expression level and the Cobb angle was found for all patients (*N* = 33) concerning the superficial muscles (*p* = 0.11), the deep concave (*p* = 0.86), or the deep convex muscles (*p* = 0.22). However, once the patients were divided into subgroup presenting *rESR2* > 1 (*N* = 20) and showing *rESR2* < 1 (*N* = 13), the subgroup analysis revealed a significant correlation in the first subgroup (*rESR2* > 1): r = 0.73, *p* = 0.004 and no significant correlation in the second subgroup (*rESR2* < 1): r = −0.36, *p* = 0.27.

No significant correlation between the *ESR2* expression level and the Progression Risk Factor was found, *p* = 0.2. However, once the patients were divided as described above *(rESR2* > 1, *N* = 20 and *rESR2* < 1, *N* = 13), the subgroup analysis revealed a significant correlation in the first subgroup: r = 0.47, *p* = 0.041 and no significant correlation in the second subgroup (*rESR2* < 1): r = −0.48, *p* = 0.11.

## 4. Discussion

Using the reverse transcription followed by polymerase chain reaction, the western blot, and immunohistochemistry techniques, the estrogen receptors ESR1 and ESR2 presence were confirmed in the muscular cells of the paraspinal skeletal muscles in a cohort of children with idiopathic scoliosis. Our findings confirm the observations already published by other authors. So far, the published studies on the topic have been carried out in smaller groups: 7 patients for the Lemoine et al. [8] study, 6 patients for the Wiik et al. [9,10] studies, 14 patients for the Kudo et al. study [7], or 18 scoliotic patients for the Zamecnik et al. studies [11,12]. The current study, to the best authors’ knowledge, presents the largest published sample.

Positive immunohistochemical staining for ESR1 and ESR2 proteins was observed in the paraspinal muscle cells nuclei. The ESR1 expression was also observed in the muscle cell membrane indicating the presence of membrane-associated G-protein, sometimes referred to as a so-called third estrogen receptor (GPR30), associated with the non-genomic estrogen receptors’ action [31].

The RT-qPCR technique allowed quantifying the mRNA estrogen receptors expression level in paravertebral muscles of female adolescent patients (*N* = 35), surgically treated for idiopathic scoliosis. This study showed significantly higher levels of ESR1 expression in deep paravertebral muscles than in superficial ones. No such difference was identified for ESR2. Interestingly, the deep paravertebral muscles present the main muscle body around the spine responsible for both mobility and stability of the vertebral column. They are suspected to be involved in the mechanism of scoliosis occurrence or progression [5]. On the other hand, the back superficial muscles, even if originating from the tips of the spinous processes, belong to the shoulder girdle muscles.

In 2003, Wiik et al. reported the *ESR1* expression level to be 180 times higher than the *ESR2* level in the vastus lateralis muscle [9]. In our study, the *ESR1* to the *ESR2* difference was not as important as reported for the vastus lateralis muscle.

The asymmetry of expression of genes responsible for various relevant ligands or receptors was previously reported for the deep paravertebral muscles of patients with idiopathic scoliosis. Acaroglu et al. examined the paravertebral muscles of 18 girls and two boys; they reported a significantly higher calmodulin expression level on the convex than the concave side, while melatonin expression level was found to be similar on both sides of the curvature [32]. On the other hand, Qiu et al. examined 17 girls and three boys; they found higher melatonin receptor type 2 expression levels on the concave compared to the convex side [33]. Kudo et al. stated that the *ESR1* mRNA expression on the convex side was higher than on the concave side in the patients with IS, while the expression ratio of *NGF* and *ESR1* in the AIS group was higher than that of control subjects [7]. In turn, Zamecnik et al. do not support the difference in the ESR2 expression level in the paravertebral muscles in patients with scoliosis compared with the control group without scoliosis [11].

**Figure 4:**
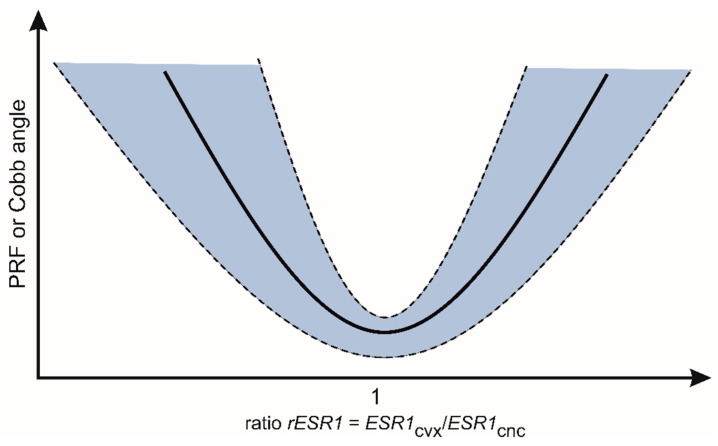
Relationship between the ESR1 expression asymmetry (*rESR1*) and two phenotype parameters: the scoliosis angle (Cobb angle) or the Progression Risk Factor (PRF). The two phenotype parameters, denoting the scoliosis magnitude and the scoliosis deterioration risk, respectively, revealed minimal values in patients presenting symmetry of *ESR1* expression (*rESR1* of about 1).

The rationale for introducing the ratio parameter (convex to concave expression ratio) is that this ratio enables the detection of asymmetry of estrogen receptors expression within the skeletal paraspinal muscles. In the case of perfect symmetry of expression, the value of the ratio (convex to concave) is equal to 1.0. In the case of a higher convex expression, the ratio value is superior to 1.0 (r > 1.0), while in the case of a higher concave expression, the ratio value is inferior to 1.0 (r < 1.0). As the estrogen receptors are involved in skeletal muscle function, one can hypothesize that the skeletal muscle function around the apical area of scoliosis could be assessed with this analysis. In the past, the asymmetrical activity of skeletal paraspinal muscles was evoked as the factor related to scoliosis onset or to scoliosis progression in the frame of the “muscular” theory of idiopathic scoliosis etiopathogenesis. Our findings seem to support such a hypothesis. In this study, both the Cobb angle and the Progression Risk Factor expressed the highest values (more severe scoliosis deformity or more progressive scoliosis) when the ratio was clearly superior or clearly inferior to 1.0 (Figure 4).

The observed asymmetry of *ESR1* expression might result from mechanical loads applied to the muscles located on either side of the spinal curvature in mechano-transduction. This phenomenon may occur in muscle cells and consists of the conversion of mechanical energy into chemical signals that can cause modification of the expression of certain genes, including the *ESR1* gene [6,15]. In 2013, Kim et al. [34] described a spatial model of the musculoskeletal system, which consisted of a simplified trunk model, lumbar spine, pelvis, and muscles combined with an optimization technique to calculate muscle forces in upright and isometric forward flexed posture. The researchers stated that when the muscles are properly activated, they maintain posture and stabilize the lumbar spine.

We report a correlation between the Cobb angle value or the PRF coefficient value against the degree of asymmetry of the *ESR1* expression level. Analysis of the relationship between phenotype-related parameters, namely the Cobb angle or the coefficient of progression (PRF) versus the asymmetry of *ESR1* expression levels showed that with increasing Cobb angle or increasing PRF value, the asymmetry between the level of *ESR1* expression on the convex side versus the concave side increased. The detected correlations were statistically significant and arranged in a specific way, as illustrated in Figure 4. The lowest values of the Cobb angle or the PRF were found for the *ESR1* ratio = 1, corresponding to symmetrical ESR1 expression.

The asymmetry of *ESR1* expression might be related to the asymmetry of muscular load on either side of scoliosis. These observations may expand our knowledge about the *ESR1* and *ESR2* expression in paravertebral muscles and the muscles’ role in the pathomechanism of scoliosis. The current state of knowledge does not allow to settle whether the muscles changes are primary or secondary to developing spine deformity.

## 5. Conclusions

Two types of estrogen receptors are present in the deep paravertebral muscles of patients with idiopathic scoliosis. Their expression was found to be asymmetrical around the apex of the principal scoliosis curvature. The expression asymmetry was revealed to be related to the magnitude of the scoliotic deformity as well as to scoliotic deformity progression. These findings may contribute to understanding the etiopathogenesis of IS in children.

## Figures and Tables

**Table 1:** Selected parameters measured in the patients.

Parameter	Values			
Age of surgery mean ± SD (range)	15 years 5 months ± 26 months (12–21 years; 142–254 months)			
Height mean ± SD (range)	160.1 ± 7.1(147–176 cm)			
C7 plumbline	7 left	7 neutral	19 right	
Angle of trunk rotation mean ± SD (range)	17.9 ± 5.7 (5–30 degrees)			
Cobb angle mean ± SD (range)	74.8 ± 19.1 (50–114 degrees)			
Apical vertebra level	3.3% Th7 or above	76.7% Th8 or Th9	20% Th10 or below	
Risser sign %	0 or 1 or 2 15%	Risser 3 18%	Risser 4 55%	Risser 5 12%

Legend: SD-standard deviation.

**Table 2:** Primers and probes used in qPCR.

Primer Name	Primer Sequence	Probe
ESR1 forward primer	5′-AATGCTACGAAGTGGGAATGAT-3′	probe #67 (Roche Cat. No. 04688660001).
ESR1 reverse primer	5′-CAAAGGTTGGCAGCTCTCAT-3′	
ESR2 forward primer	5′-GAAGCATTCAAGGACATAATG-3′	N/A
ESR2 reverse primer	5′-TCCCACTTCGTAACACTTC-3’	
HPRT forward primer	5′-TGAAGAGCTATTGTAATGACCAGTC-3′	N/A
HPRT reverse primer	5′-CAAATCCAACAAAGTCTGGC-3′	

Legend: N/A-not applicable.

**Table 3:** Thermal profile for genes quantification.

Cycles	Analysis Mode		UPL Probes *ESR1* and *HPRT*	SybrGreen I	
				*ESR2*	*HPRT*
1	Pre-Incubation		95 °C, 10 min		
45	amplification and quantification	denaturation	95 °C, 10 s		
		annealing	60 °C, 30 s	55 °C, 5 s	54 °C, 5 s
		elongation		72 °C, 11 s	72 °C, 9 s
		fluorescence data acquisition	72 °C, 1 s	at the end of the elongation step	
1	melting curve	denaturation	none	95 °C, 0 s	
		annealing		65 °C, 5 s	
		denaturation with continuous fluorescence data acquisition		95 °C, rampling 0.1 °C/s	
1	cooling		40 °C, 30 s		

Legend: UPL-universal ProbeLibrary; *HPRT*-hypoxanthine-guanine phosphoribosyltransferase reference gene; *ESR1*-estrogen receptors type 1 gene; *ESR2*-estrogen receptors type 2 gene.

**Table 4:** *ESR1* and *ESR2* expression levels in superficial and deep back muscles in 35 cases.

Muscle Group	Minimum	Maximum	Mean	SD
*ESR1*				
superficial	48.0 × 10^−6^	67.2 × 10^−5^	24.4 × 10^−5^	15.1 × 10^−5^
deep concave	97.0 × 10^−6^	11.5 × 10^−4^	41.3 × 10^−5^	28.4 × 10^−5^
deep convex	82.0 × 10^−6^	30.0 × 10^−4^	48.2 × 10^−5^	52.9 × 10^−5^
*ESR2*				
superficial	21.0 × 10^−6^	80.0 × 10^−3^	16.7 × 10^−3^	35.91 × 10^−3^
deep concave	12.1 × 10^−4^	14.0 × 10^−2^	20.72 × 10^−3^	27.27 × 10^−3^
deep convex	94.6 × 10^−5^	50.0 × 10^−3^	15.57 × 10^−3^	12.63 × 10^−3^

Legend: SD-standard deviation.

## Data Availability

The datasets used and analyzed during the current study are available from the corresponding author on reasonable request.
